# A novel method of clinical first tarsometatarsal joint hypermobility testing and radiologic verification

**DOI:** 10.1007/s00508-020-01705-x

**Published:** 2020-07-02

**Authors:** Martin Ornig, Sebastian Tschauner, Patrick Lukas Holweg, Gloria Maria Hohenberger, Gerhard Bratschitsch, Andreas Leithner, Lukas Leitner

**Affiliations:** 1 Department of Orthopedics and Trauma, Medical University of Graz, Auenbruggerplatz 5, 8036 Graz, Austria; 2 Department of Radiology, Medical University of Graz, Graz, Austria

**Keywords:** Hallux valgus, Tarsometatarsal joint, Instability, Lapidus, Clinical testing

## Abstract

**Background:**

First tarsometatarsal joint (TMT-1) hypermobility might cause hallux valgus deformity (HV), and recurrence following surgical correction. Anatomic findings, indicating tibialis anterior tendon (TAT) involvement in TMT‑1 stabilization, led to the development of cross-glide test allowing clinical TMT‑1 stability testing. Cross-glide test function was evaluated in anatomical specimens and in the clinical setting, compared to simulated weight-bearing computer tomography (CT) analysis.

**Methods:**

Cross-glide test was evaluated in 6 healthy lower leg specimens before and after TAT transection. Clinical testing was performed prospectively in 36 feet (6 controls, 21 HV, 9 recurrent HV); consecutive weight-bearing CT analysis was performed. Results from clinical testing were compared to CT analysis.

**Results:**

TMT‑1 instability significantly increased in anatomic specimens following TAT transection (*p* = 0.009). In the clinical setting, all healthy feet were cross-glide test negative, 62% of HV cases and all recurrent HV feet were positive. In the CT analysis- Compared to controls the HV cases revealed significantly increased MT‑1 internal rotation (*p* = 0.003) and decreased dorsal angle (*p* = 0.002), considered as collapsing forefoot signs; HV recurrent cases revealed similar results. Positive cross-glide tested cases revealed increased MT‑1 internal rotation values (*p* < 0.001) and dorsal angle values (*p* < 0.001) in CT analysis. Strikingly, cross-glide test positive HV cases revealed significantly increased internal TMT‑1 rotation (*p* = 0.043) in CT analysis, and HV and IMT (intermetatarsal) angle were significantly higher (*p* = 0.005, *p* = 0.006). 15 HV recurrence cases, treated with TMT‑1 arthrodesis, revealed no recurrence during follow-up.

**Conclusion:**

Cross-glide test allows reliable clinical TMT‑1 instability testing, via TAT tension, and is less laborious than CT analysis. We recommend TMT‑1 arthrodesis in cases with instability in clinical testing, to avoid HV recurrence.

**Video online:**

The online version of this article contains one video. The article and the video are online available (10.1007/s00508-020-01705-x). The video can be found in the article back matter as “Electronic Supplementary Material”.

## Introduction

Hallux valgus (HV) deformities can be found in more than 35% of the population aged over 65 years [1], a multitude of different surgical treatment procedures suggests an incomplete understanding of the underlying process [2, 3].

Hypermobility of the first tarsometatarsal joint (TMT-1) can occur concomitantly with HV deformity. Since the clinical diagnosis is difficult, the role of TMT‑1 instability on development of HV deformity is controversially discussed [3, 4]. Persisting TMT‑1 hypermobility, following surgical HV correction via distant MT‑1 osteotomy, might be insufficient in solving the underlying problem, and consequently cause HV recurrence. Therefore, the inclusion of TMT‑1 arthrodesis (Lapidus procedure) during HV correction, might be the most sufficient approach in such cases [5, 6].

Triggered by this problem, several clinical TMT‑1 hypermobility testing methods have been described in literature [7], but a residual subjective component remained in their quantification—reproducible clinical quantification was considered lacking so far [8–10]. More recently, several methods of TMT‑1 hypermobility quantification via weight-bearing computer tomography (CT) analysis were described for the first time providing measurable, reproducible results [2, 3, 11].

Since CT scan analysis is correlated with increased radiation exposure to patients, and many patients are hardly able to perform this burdensome testing due to their constitution, reliable clinical testing of TMT‑1 stability, which is reproducible via CT measurement, would offer a straightforward solution.

Furthermore, recent anatomical research suggests additional mechanical stabilization of TMT‑1 via the tibialis anterior muscle tendon (TAT), which was shown inserting at medial cuneiform (MC) and, as novel data suggest, additionally first metatarsal (MT-1) base [12–14]. This newly recognized anatomical feature might be relevant for TMT‑1 stabilization and hypermobility and should therefore be included in the clinical testing of TMT‑1.

The aim of this study was the evaluation of a novel clinical TMT‑1 instability testing method called cross-glide test (1) via functional assessment of TMT‑1 and TAT in anatomic specimens and (2) subsequent examination of clinical cases, where a correlation with results from simulated weight-bearing CT, concerning validity should be performed.

## Methods

### Cross-glide test

**Fig. 1:**
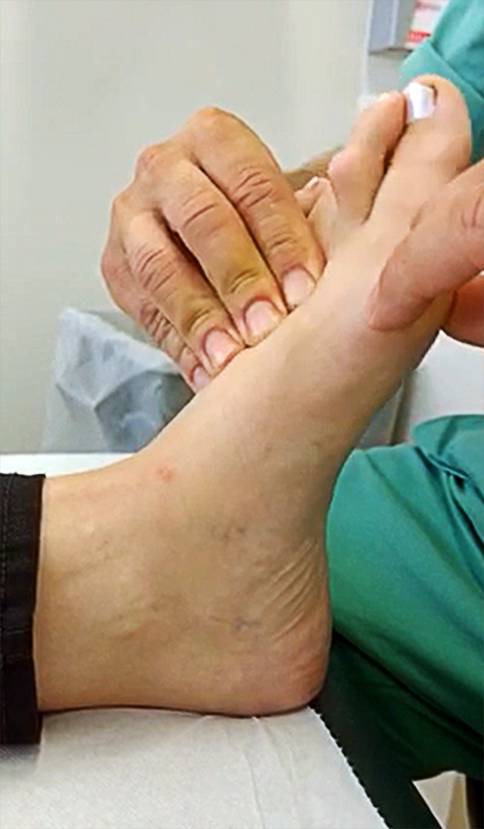
Manual examination of first ray mobility using the cross-glide test. The ankle should be held in a neutral position during the examination, in maximum eversion of the foot whilst the lesser metatarsals are fixed and the first metatarsal is moved in a dorsoplantar direction by the examiner (also see Supplementary video)

The cross-glide test is a clinical testing method for TMT‑1 instability. Similar to earlier described tests, fixation of the ankle in a neutral position, which is maintained during the whole test, is mandatory [15], in maximum eversion of the foot—presenting the main novelty of the test. Sagittal and transverse motion (grasping test, clinical squeezing test) of the TMT‑1 is then performed as earlier described (Fig. 1, supplementary video) [16, 17]. The index finger and thumb of one hand are used to grasp the lesser metatarsals (fixating talonavicular joint and naviculocuneiform joint). The other hand grasps the first metatarsal and moves the first metatarsal head in a dorsoplantar direction. The ankle should be held in a neutral position during the examination. Test result was either assigned as positive (clear hypermobility of TMT-1) or negative (rigid TMT-1) according to the observer’s clinical impression.

### Study population and data

This study was approved by the Institutional Ethical Review Board of the Medical University of Graz (Graz, Austria; reference number: 29-099 ex 16/17). All experiments were performed in accordance with relevant guidelines and regulations. Anatomic specimens were obtained from voluntary donors, who consented during their lifetime to donate their body for research and teaching purposes to the Center for Anatomy and Cell Biology at our Medical University. Informed consent was obtained from all clinical participants. Hallux valgus feet were defined according to common radiologic criteria, pes planus deformity and other rear midfoot and forefoot deformities were defined as exclusion criteria. Patient demographic characteristics (age, sex, body mass index, BMI) were retrieved from our hospital database system.

### Lower leg specimens

The study sample included 6 lower extremities gained from human adult cadavers, embalmed using Thiel’s method [18]. Selection criteria were absence of HV and obvious signs of earlier surgical interventions, as indicated by local scars, or other pathologies.

**Fig. 2:**
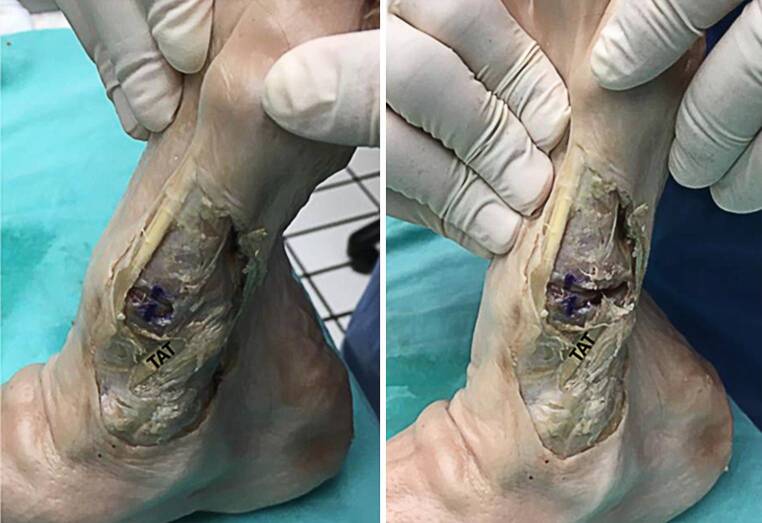
**a** Cross-glide test on intact tibialis anterior tendon (TAT) inserting at first tarsometatarsal joint (TMT‑1) in anatomical specimen as earlier described [12] **b** Cross-glide test after transection of TAT in TMT‑1 area in the same specimen, resulting in instability, as indicated by *blue marks*

Skin and subcutaneous tissue were removed from the TMT‑1 area with a scalpel, all muscles and tendons were preserved. The tibialis anterior tendon (TAT) and its corresponding bony insertions were explored and documented by photograph according to a standardized protocol. TMT‑1 stability was tested using clinical squeeze test and cross-glide test in maximum eversion of the foot. This procedure was repeated after transection of TAT on TMT‑1 level; all measurements were documented (Fig. 2).

### Clinical and radiologic testing

**Fig. 3:**
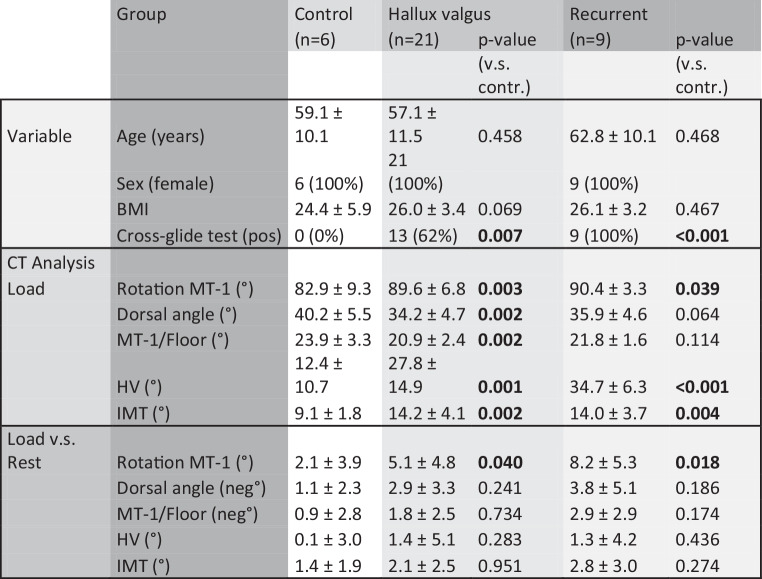
Comparison between healthy controls, hallux valgus and recurrent hallux valgus group concerning clinical data, simulated weight-bearing computer tomography (CT) analysis, and simulated weight-bearing CT analysis compared to non-weight-bearing CT analysis

In this study 36 feet from 18 female patients were prospectively selected from our foot specific surgical outpatients’ clinic between 11/2018 and 05/2019 and assigned into groups (6 healthy controls, 21 HV, 9 recurrent HV) according to their clinical and radiologic testing result (Fig. 3).

Cases were tested using the cross-glide test independently by an experienced foot and ankle surgeon (M.O.) and a resident surgeon (L.L.), and the measurements were documented. To allow radiological comparison between clinically/cross-glide testing positive and negative HV cases, which we considered most relevant for our hypothesis, we included all available HV patients with positive cross-glide test who agreed to participate in our study (*n* = 13).

**Fig. 4:**
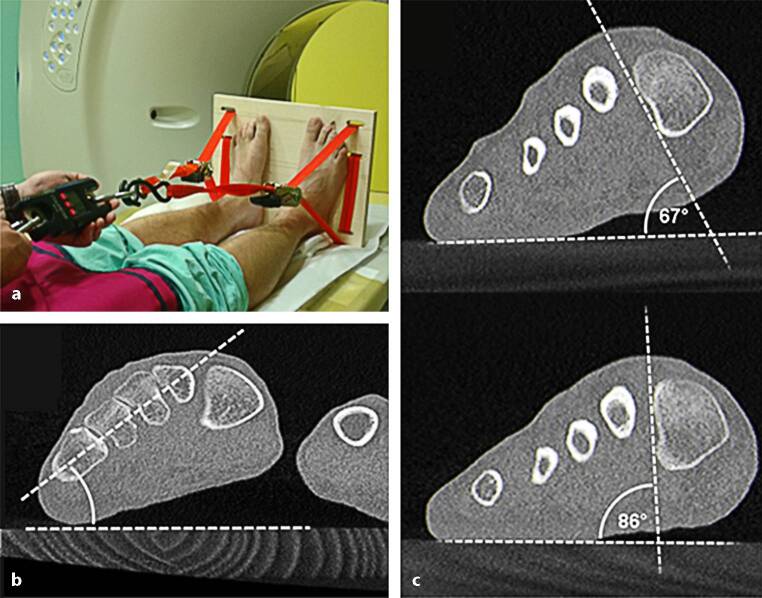
**a** Simulated weight-bearing computer tomography (CT) scan was performed on the loading device with the lower limbs extended and the ankle joint in a neutral position. Analysis of CT scans was performed measuring dorsal angle (**b**) and MT‑1 rotational angle as shown here in non-weight-bearing (**c**, upper) and weight-bearing position (**c**, lower) as earlier described [3, 11]

The CT scans were performed as earlier described in detail by [11, 19], in short form. A non-weight-bearing CT scan was first performed whilst feet were placed in neutral position on the loading device with the lower limbs extended and the ankle joint in a neutral position, followed by a simulated weight-bearing CT scan in unaltered position, with a load equal to body weight applied to the loading device (Fig. 4a). This method is called simulated weight bearing in the literature, since it is not a CT in a standing weight bearing position, but simulates this situation.

The examination protocol for each of the two examination series contained fixed exposure parameters of 120 kV (kilovoltage) and 50 mA (tube current) with collimation of 0.5 mm for all 320 active detector rows, covering 16 cm in the z‑axis and resulting in a volumetric computed tomography dose index (CTDIvol) of 4.8 mGy each. The effective dose of the whole study was about 0.13 mSv.

A blinded radiologist with 7 years of experience in musculoskeletal imaging analyzed the data in various sectional planes (multi-planar reformation, MPR) and different slice thicknesses.

Analysis was performed measuring hallux-valgus (HV) angle, intermetatarsal (IMT) angle, MT‑1 rotational angle and dorsal angle as earlier described by ([3, 11]; Fig. 4b, c).

During the study period 15 cases of recurrent HV and clinical TMT‑1 instability were treated with TMT‑1 arthrodesis and included in a clinical and radiological follow-up.

### Statistical methods

SPSS Statistics 20 (IBM, Amonk, NY, USA) was used for data analysis. Statistical analysis was performed using χ^2^-test for comparison of categorical parameters, t‑test for comparison of continuous normally distributed parameters and Spearman’s correlation coefficient for calculation of correlations. A two-sided *p*-value <0.05 was considered statistically significant.

## Results

TMT‑1 appeared strained in all anatomic specimens (*n* = 6) when cross-glide test was performed (2.4 ± 0.5 mm). After TAT had been dissected on TMT‑1 level, TMT‑1 appeared significantly more unstable (4.2 ± 0.8 mm; *p* = 0.009) when testing was repeated (Fig. 2) indicating TAT to be an important structure in the clinical testing via cross-glide test.

Consequently 36 feet (all female), 6 healthy controls, 21 hallux valgus cases according to their HV and IMT angle, and 9 recurrent HV cases, where MT‑1 correction osteotomy had been performed before, were selected; further division of HV group was performed according to clinical testing result (cross-glide test positive and negative).

Results of comparison between healthy controls and HV/recurrent HV group are shown in detail in (Fig. 3): Whilst age and BMI where comparable in all groups, and all patients were female, HV and IMT angle were significant larger in HV group (*p* < 0.001) and recurrent HV group (*p* < 0.001) compared to controls. Cross-glide test was negative in all control cases, whilst it was positive in 62% (*n* = 13) of HV cases. Interestingly, cross-glide-test was positive in 100% of recurrent HV cases, indicating unstable TMT‑1 joint in all recurrent cases. Result from cross-glide test was concordant in 97% of all cases between experienced surgeon and resident surgeon.

In the simulated weight-bearing CT analysis, HV cases revealed significantly increased MT‑1 internal rotation (*p* = 0.003) and dorsal angle (*p* = 0.002) compared to control group, MT‑1 internal rotation significantly increased compared to non-weight-bearing analysis (*p* = 0.04) and can be considered as radiologic sign for forefoot instability. Recurrent HV cases revealed similar signs of collapsing forefoot (Fig. 3).

**Fig. 5:**
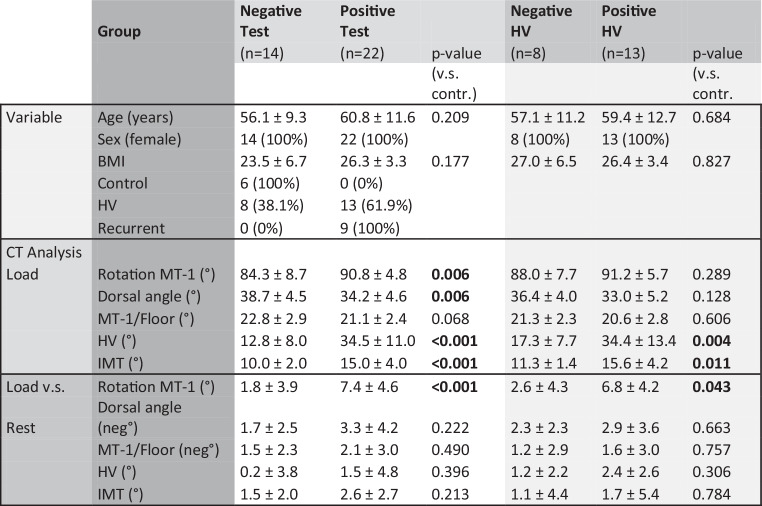
Comparison between cross-glide test negative and positive cases, and subgroup analysis in hallux valgus group, concerning clinical data, simulated weight-bearing computer tomography (CT) analysis, and simulated weight-bearing CT analysis compared to non-weight-bearing CT analysis. *HV* hallux valgus

Results of comparison between cross-glide test positive and negative group are shown in detail in (Fig. 5): Whilst age and BMI where comparable in all groups, and all patients were female, cases with positive cross-glide test revealed significantly increased MT‑1 internal rotation values (*p* < 0.001) and dorsal angle values (*p* < 0.001) simulated in weight-bearing scans. The MT‑1 internal rotation also significantly increased compared to non-weight-bearing analysis (*p* < 0.001) (Fig. 5), radiologically indicating increased forefoot instability in cross-glide test positive cases.

Strikingly, subgroup analysis of HV cases tested positive (unstable TMT-1) and HV cases with negative cross-glide test (stable TMT-1) revealed, that cross-glide test positive cases had significantly increased internal TMT‑1 rotation in the CT scan, when weight-bearing was applied (*p* = 0.043) (Fig. 5). Furthermore, HV and IMT angle were significantly higher (*p* = 0.005 and *p* = 0.006) in positively tested HV cases compared to negatively tested HV-cases (Fig. 5).

Resulting from our preliminary experimental results, TMT‑1 instability in HV recurrent cases was used as indication for TMT‑1 arthrodesis between 2017 and 2019 at our institution in 15 patients, with no reported recurrent HV case in this group during follow-up (19.9 ± 6.6 months).

## Discussion

Clinical cases of recurrent HV following MT‑1 osteotomy, led us on the track of TMT‑1 instability in certain cases, where isolated distal MT‑1 correction seems insufficient.

TMT‑1 instability has earlier been in the focus in the context of HV correction but discussed controversially [4–6]. A reproducible clinical proof that HV is associated with increased TMT‑1 instability in certain cases, was missing so far [17], which we attempted to show for the first time, combining recent anatomical findings with clinical testing and simulated weight-bearing CT.

As we could show in our anatomical specimen as a first step, TMT‑1 instability seems to be a manifestation of TAT insufficiency, which can be detected by our newly developed cross-glide test. The anatomical relevance of TAT in the functional integrity of TMT‑1 has recently been demonstrated by several authors [12–14]. The cross-glide test, as presented in this manuscript, involves this influence of TAT on TMT‑1 stability via maximum eversion. The transection of TAT on TMT‑1 level in anatomic specimen, immediately caused a positive clinical testing.

In the next step simulated weight-bearing CT analysis was performed of all clinical cases, where we could detect increased instability criteria of TMT‑1 in HV cases, as earlier described [20], which were also present in HV recurrent cases, but missing in healthy controls (Fig. 3), supporting the validity of this method. When cases were divided into cross-glide test positive and negative cases, these radiologic instability criteria of TMT‑1 were significantly increased in positive cases, indicating radiologic reproducibility of our clinical results (Fig. 5).

Strikingly, in the HV subgroup, cases with clinical instability testing of TMT‑1 also revealed significantly increased rotational instability of MT‑1 in the simulated weight-bearing CT analysis (Fig. 5), underlining the ability of cross-glide test to identify HV cases, where isolated distal MT‑1 correction might be insufficient. Our clinical experience that unstable TMT‑1 joint is a risk factor for HV recurrence, is supported by our findings: All included HV recurrence cases revealed signs of TMT‑1 instability, clinically and radiologically.

Cross-glide test furthermore seems to be well reproducible between different observers, even by clinicians with less clinical experience, as our results show, whilst it is less laborious for patients and physicians compared to simulated weight-bearing CT testing.

From our clinical practice, we recommend primary stabilization of TMT‑1 in HV cases with positive cross-glide test in order to avoid recurrent HV formation, whilst stable cases can be treated with first-row osteotomy according to recent guidelines [21]. If joint-preserving surgery is preferred (e.g. young age, moderate IMT angle <15°, pain at the pseudoexostosis as main problem), thorough patient education concerning HV recurrence risk should be documented. This is supported by our presented cases with recurrent HV formation with clinical and radiologic TMT‑1 instability, which have been revised using TMT‑1 arthrodesis (Lapidus procedure), with stable first ray and no evidence for HV recurrence during follow-up. Finally, clinical and radiologic long-term follow-up will clarify the question whether this practice results in lower HV recurrence rate and comparable quality of life and activity outcome.

A limitation of our study is that the study center was a university hospital with a highly specialized outpatients’ clinic. Since standard HV cases are most commonly treated outside specialized centers, prevalence of TMT‑1 instability will be grossly overrepresented in our sample. This leads to the impression that we recommend TMT‑1 arthrodesis in up to 62% of our HV cases, since they are clinically unstable, which is a consequence from the mentioned selection bias. The study population is therefore not representative for a standard HV population, where the proportion of TMT‑1 instability is significantly lower.

## Conclusion

The newly presented cross-glide test seems to be a reliable clinical testing method for TMT‑1 instability, via TAT tension; whilst it is less laborious for patients and physicians compared to radiologic testing. We recommend TMT‑1 arthrodesis (Lapidus procedure) as a preferable surgical treatment in cases with positive cross-glide test/TMT‑1 instability, to reduce the possibility of HV recurrence.

## Caption Electronic Supplementary Material

This video shows the manual examination of first ray mobility using the cross-glide test. The ankle should be held in a neutral position during the examination, in maximum eversion of the foot whilst the lesser metatarsals are fixed and the first metatarsal is moved in a dorsoplantar direction by the examiner

## Abbreviations

BMI: Body mass index
CT: Computer tomography
HV: Hallux valgus deformity
TAT: Tibialis anterior tendon
TMT‑1: First tarsometatarsal joint
